# Proteome-wide identification of S-sulfenylated cysteines reveals metabolic response to freezing stress after cold acclimation in *Brassica napus*

**DOI:** 10.3389/fpls.2022.1014295

**Published:** 2022-09-29

**Authors:** Liangqian Yu, Zezhang Dai, Yuting Zhang, Sidra Iqbal, Shaoping Lu, Liang Guo, Xuan Yao

**Affiliations:** ^1^ National Key Laboratory of Crop Genetic Improvement, Huazhong Agricultural University, Wuhan, China; ^2^ Department of Plant Breeding and Genetics, University of Agriculture, Faisalabad, Pakistan; ^3^ Hubei Hongshan Laboratory, Wuhan, China

**Keywords:** redox regulation, redoxome, sulfenylation, freezing stress, proteomics, *Brassica napus*

## Abstract

Redox regulation plays a wide role in plant growth, development, and adaptation to stresses. Sulfenylation is one of the reversible oxidative post-transcriptional modifications. Here we performed an iodoTMT-based proteomic analysis to identify the redox sensitive proteins *in vivo* under freezing stress after cold acclimation in *Brassica napus*. Totally, we obtained 1,372 sulfenylated sites in 714 proteins. The overall sulfenylation level displayed an increased trend under freezing stress after cold acclimation. We identified 171 differentially sulfenylated proteins (DSPs) under freezing stress, which were predicted to be mainly localized in chloroplast and cytoplasm. The up-regulated DSPs were mainly enriched in photosynthesis and glycolytic processes and function of catalytic activity. Enzymes involved in various pathways such as glycolysis and Calvin-Benson-Bassham (CBB) cycle were generally sulfenylated and the metabolite levels in these pathways was significantly reduced under freezing stress after cold acclimation. Furthermore, enzyme activity assay confirmed that the activity of cytosolic pyruvate kinase and malate dehydrogenase 2 was significantly reduced under H_2_O_2_ treatment. Our study provides a landscape of redox sensitive proteins in *B. napus* in response to freezing stress after cold acclimation, which proposes a basis for understanding the redox regulation in plant metabolic response to freezing stress after cold acclimation.

## Introduction

Post-translational modifications (PTMs) of proteins are necessary for formation of mature proteins that ultimately accumulate in plant cells (Smith and Kelleher, 2013). PTMs can occur spontaneously or enzymatically, which depend on the physico-chemical properties of reactive amino acids and their environments such as pH, redox status, and metabolites (Ryšlavá et al., 2013). It is also an effective way that cells can rapidly regulate the activity, intracellular localization, stability of proteins and protein-protein interactions during metabolism, development and stress responses, representing some of the quick plant responses to environmental changes (Huber and Hardin, 2004; Friso and van Wijk, 2015; Millar et al., 2019). There are seven known oxidative modifications of cysteine, five of which are reversible (Held and Gibson, 2012). Among these reversible oxidations of cysteine, S-nitrosylation (SNO) and S-sulfenylation (SOH) play a significant role in regulating cellular signaling in response to redox imbalance. SNO is a covalent addition of nitroso group (-NO) onto the reduced thiol of cysteines. The common endogenous sources of nitroso group include the nitric oxide synthase (NOS) family of enzymes (Foster et al., 2009). Sulfenylation on thiol (SH) of cysteine by H_2_O_2_ is a common spontaneous form of PTMs to produce sulfenic acid (SOH), which is transiently stable and can be further overoxidated to sulfinic (SO_2_H) and sulfonic (SO_3_H) acids (Antelmann and Helmann, 2011; Paulsen and Carroll, 2013). Cysteine sulfenate is also an important intermediate in the formation of disulfides within or between proteins (Hogg, 2003). This kind of reversible oxidation can protect cysteine residues against irreversible overoxidation to the sulfinic and sulfonic acids by formation of disulfides with proteins, glutathione, or other redox PTMs like S-sulfyhydration (R-SSH) (Hochgräfe et al., 2007; Zivanovic et al., 2019). A systematic and exhaustive approach for identification of oxidized cysteine residues is defined as a redox proteomic or redoxome analysis (Chiappetta et al., 2010). 2DE separation-based and MS-based methods have been applied and developed in plant research for over a decade, which would help establish the network of cysteine residue oxidation and provide clues to uncover its mechanism (Wang et al., 2012; Liu et al., 2014; Zhu et al., 2014; Huang et al., 2019).

ROS also play a role in the regulation of metabolic fluxes during stresses to prevent the oxidative damage on cell (Choudhury et al., 2017). Metabolic pathways in plant are sensitive to environmental changes which mainly manifest in inhibited photosynthesis, and metabolic imbalances can promote the generation and accumulation of reactive oxygen species (ROS) (Suzuki et al., 2012; Poonam et al., 2015). Moreover, it is a common strategy for plants to survive in adversity by slowing down the growth, and this so-called ‘active’ growth inhibition is supposed to be obtained through cell signal transduction (Zhang et al., 2020). H_2_O_2_ functions as a signaling molecule and its role in controlling plant growth and development has been given more consideration (Htet et al., 2019; Nazir et al., 2020). It has been revealed that H_2_O_2_ improves the tolerance of plants to both drought and salt stresses, which enables the plants to memorize and rapidly activate early signals during re-exposure to the stresses (Ellouzi et al., 2017). Modification of cysteine thiols by redox substances has been known as an important signaling mechanism for conveying cellular responses (Finkel, 2003; Tonks, 2005). Both biotic and abiotic stresses can cause ROS production, leading to modification of cysteine oxidation profiles (Suzuki et al., 2012; McConnell et al., 2019; Mhamdi, 2019). Different abiotic stresses and their combinations probably cause the formation of different ROS signatures *via* different ROS sensors, which can create a stress-specific signal for plant adaptation to stresses (Choudhury et al., 2017). Freezing stress is one of the major abiotic stress factors affecting the over-wintering rate and yield of rapeseed (Sun et al., 2010). Freezing injury is the most immediate injury for leaves which are directly exposed to air temperatures (Cavender-Bares, 2007). Understanding the physiological and molecular mechanism of winter rapeseed (*Brassica napus* L.) in response to freezing stress is essential for the improvement of cold and freezing tolerance of *B. napus*. However, little is known about the complex regulatory mechanism of *B. napus* response to freezing stress.

Oxidized metabolites and proteins have been revealed to function in signaling under oxidative conditions (Mueller and Berger, 2009; Noctor and Foyer, 2016). In addition to modified proteins and metabolic fluxes, ROS and RNS induced by stresses also interact with plant hormones (Freschi, 2013; Mur et al., 2013; Choudhury et al., 2017). H_2_O_2_ induces the reversible oxidation of the BRASSINAZOLE-RESISTANT1 (BZR1) transcription factor, which mediates BR signaling to regulate plant development (Tian et al., 2018). A newly identified H_2_O_2_ receptor, HPCA1, encoding an LRR receptor kinase, activates Ca^2+^ channels by its oxidized extracellular cysteine residues to further regulate stomatal closure (Wu et al., 2020). The transcription factor bZIP68 plays an important role in plant growth and the adaption to stresses. Its inactivation can improve the resistance to stresses by repressing plant growth, which is also controlled by the extent of its oxidation (Li et al., 2019). Multiple evidences suggest that the oxidative modification of cysteine by accumulated ROS is an important pattern of signal transduction for plants in adaptation to stresses. However, our knowledge of distribution and function of redox-sensitive proteins in plants remains limited. Therefore, it is necessary to carry out thorough and accurate identification of the endogenous oxidation modified proteins in plants.

Proteomic techniques have been widely used in the identification of PTMs, in which iodoTMT strategy, has been proved to be precise and sensitive enough to detect and quantify endogenous cysteine oxidation levels in a proteome-wide scale (Wojdyla et al., 2015). Applying this iodoTMT-based method we identified the *in vivo* sulfenylated cysteine sites in *B. napus* under freezing stress after cold acclimation. Finally, we obtained 1,372 sulfenylated sites in 714 proteins, and 252 sites in 171 proteins were differentially sulfenylated under freezing stress after cold acclimation. The glycolysis and photosynthesis processes were potentially regulated by redox-mediated PTMs because multiple key enzymes in these pathways were differentially sulfenylated and the relevant primary metabolites were significantly reduced under freezing stress after cold acclimation. These findings provide a basis and inspiration for understanding the redox regulation in plant response to freezing stress. This is even more significant for winter rapeseed production, which is threatened by cold and freezing injury during the long winter.

## Materials and methods

### Plant growth and stress condition

*B. napus* cultivar zhongshuang11 seedlings were kept in a growth room under 22°C/(16 h light: am7:00-pm23:00)/18°C (8 h dark: pm23:00-am7:00) with 200 µmol s^-1^ m^-2^ light intensity for two weeks. After cold adaptation at 4°C under the same photoperiod condition and light intensity for one week, the plants were subjected to freezing stress at -4°C. Leaf samples for proteomic analysis were collected after a 4 h-freezing treatment and immediately quick-frozen with liquid nitrogen. The plants grown in room temperature were taken as control.

### Experimental design and rationale

Three replicates were set for both control and treated samples. The intensity data obtained from mass spectrometer were normalized by ‘mean normalization’ method first. The iodoTMT-128 and iodoTMT-129 labelled peptides after arsenite reduction were considered as sulfenylated (R-SOH) peptides, while the TMT-126 and iodoTMT-127 labelled sites were considered as total cysteines after TCEP reduction in this study. The reductant TCEP can reduce blocked thiols and reversible modifications including sulfenylation. After that, we can label the total cysteines. While the sulfenylated cysteines (R-SOH) can be labeled after being specifically reduced by arsenite. The two reducing agents were added into fractions separated from the same sample for marking the reduction and oxidation states of thiols in the same sample. The ratio of normalized intensity of iodoTMT-128 to iodoTMT-126 (control) and iodoTMT-129 to iodoTMT-127 (stress) was calculated to characterize the sulfenylation level of sulfenylated cysteines, which was defined as %R-SOH (Cys-SOH/Cys-total), in a way that the interference of the sulfenylation level caused by changes in protein abundance can be eliminated. The protein abundance was characterized by normalized MS data of peptides labelled by iodoTMT-126 and iodoTMT-127 under control and salt stressconditions, respectively. The ratio of %R-SOH and protein abundance under stress condition to that under control were calculated. Both the differentially sulfenylated proteins and differentially expressed proteins were identified by a threshold of 1.2-fold change for for up-regulation and 0.83-fold change for for down-regulation. Three biological replicates were used for the calculation of protein abundance and sulfenylation level. A *p-*value ≤ 0.05 was considered statistically significant.

### DAB staining

3,3´-Diaminobenzidine (DAB) staining was performed with some modifications to test the H_2_O_2_ level under freezing stress after cold acclimation, according to the procedure described previously (Daudi and O'Brien, 2012). Briefly, the fresh leaves were immersed in DAB solution (1 mg mL^-1^ DAB in 10 mM Na_2_HPO_4_ and 0.05% Tween 20). Samples were vacuum-infiltrated for 30 min and then incubated for 8 h at 25°C in the dark with gentle shaking at 50 rpm. For decoloring, samples were incubated in ethanol at 95°C for 10 min and subsequently kept in 75% ethanol at room temperature for visualization. The color intensity of stained leaves was quantified using ImageJ software. The experiment was repeated for three biological replicates.

### Protein extraction

Proteins were extracted with a lysis buffer containing 8 M urea, 1% Triton-100, 1% protease inhibitor, and 50 mM S-Methyl methanethiosulfonate (MMTS) was added to the buffer for free thiols blocking (Wojdyla et al., 2015), followed by sonication for 5 min with pulse durations of 3s on/5s off at 300W for three times on ice using a high intensity ultrasonic processor (Scientz-5T, China). The remaining debris was removed by centrifugation at 20,000 g at 4°C for 10 min. The protein was then precipitated with cold 20% trichloroacetic acid (TCA) (Sigma, USA) for 2 h at -20°C. After centrifugation at 12,000 g at 4°C for 10 min, the supernatant was discarded. The pellet was washed with cold acetone for three times and finally dissolved in a buffer containing 50 mM ammonium bicarbonate and 8 M urea (pH 8.0). Protein concentration was determined with BCA kit (Beyotime biotechnology, China).

### IodoTMT labeling and trypsin digestion

Control and freezing treated samples were reduced by 10 mM Tris (2-carboxyethyl) phosphine (TCEP) and arsenite for 1 h at 37°C in darkness, respectively. The peptides were then labeled according to the manufacturer’s protocol for the iodoTMT kit (Thermo, USA). Briefly, TMT reagent was thawed and reconstituted in methyl alcohol before incubated with protein. Reactions were quenched by adding 20 mM dithiothreitol (DTT) and incubation for 15 min. Samples were pooled together and precipitated with 4 volumes of cold acetone overnight at -20°C. Pellets were washed three times with cold acetone to remove excess iodoTMT™ reagents and desalted using Strata X C18 SPE column (Phenomenex, USA) and dried by vacuum centrifugation.

The protein was digested by trypsin according to the method previously described (Li et al., 2018). After redissolved in 8 M urea, the labeled proteins were reduced with 10 mM DTT (Sigma, USA) for 1 h at 37°C and alkylated with 20 mM iodoacetamide (IAM) (Sigma, USA) for 45 min at room temperature in darkness. Samples were diluted with 50 mM ammonium bicarbonate to reduce urea concentration below 2 M and then were digested with trypsin (Sigma, USA) by a first overnight digestion at 1:50 trypsin-to-protein mass ratio and a second 4 h-digestion at 1:100 mass ratio. Peptide solution after digestion was desalted and vacuum-dried.

### HPLC fractionation and anti-TMT™ enrichment

Peptides were fractionated by high pH reverse-phase HPLC using Thermo Betasil C18 column (5 μm particles, 10 mm ID, 250 mm length). Briefly, peptides were first separated with a gradient of 8% to 32% acetonitrile (pH 9.0; Fisher Chemical, USA) over 60 min into 60 fractions and then were combined into 6 fractions. To enrich labeled peptides, tryptic peptides dissolved in IP buffer (150 mM NaCl, 250 mM Tris-HCl, pH 7.5) were incubated with pre-washed anti-TMT antibody beads (Thermo, USA) at 4°C overnight with gentle shaking. Peptides were enriched using anti-TMT antibody and four different labels were used for the labeling of R-SH (TMT-126 and TMT-127) and R-SOH (TMT-128 and TMT-129) in control and freezing treated samples, respectively. Then the beads were washed for four times with IP buffer and twice with ddH_2_O. The bound peptides were eluted from the beads with 0.1% trifluoroacetic acid (TFA). Finally, the eluted fractions were desalted and vacuum-dried for LC-MS/MS Analysis.

### LC-MS/MS analysis

The tryptic peptides dissolved in solvent A (0.1% formic acid and 2% acetonitrile in water) were directly loaded onto a reversed-phase analytical column (15 cm length, 75 μm i.d.). The gradient was comprised of an increase from 6% to 22% of solvent B (0.1% formic acid and 90% acetonitrile in water) for 60 min, 22% to 35% for 22 min and climbing to 80% in 4 min then holding at 80% for the last 4 min, all at a constant flow rate of 350 nL min^-1^ on an EASY-nLC 1000 UPLC system (Thermo, America).

The peptides were subjected to NSI source followed by tandem mass spectrometry (MS/MS) in Orbitrap Fusion™ Tribrid™ (Thermo, USA) coupled online to the UPLC. Intact peptides were detected in the Orbitrap at a resolution of 60,000. Peptides were selected for MS/MS using NCE setting as 35. Ion fragments were detected in the Orbitrap at a resolution of 15,000. MS/MS scans were acquired in a data-dependent procedure. For analysis on the Orbitrap, a full MS scan was followed by 20 MS/MS scans on the top 20 precursor ions above a threshold intensity greater than 5 × 10^3^ in the MS survey scan with 15 s dynamic exclusion. The electrospray voltage was applied at 2.0 kV. An automatic gain control (AGC) target value of 5 × 10^4^ was used to prevent overfilling of the orbitrap. For MS scans, the m/z scan range was 350 to 1,550. Fixed first mass was set as 100 m/z.

### Western blot analysis of labeled proteins

Western blot was performed to detect the labeled proteins and 4 μg proteins of each sample were separated by discontinuous SDS-PAGE. The separated proteins were electrophoretically transferred onto a nitrocellulose membrane and then detected using a TMT monoclonal antibody (25D5) as the primary antibody (Thermo Fisher Scientific, catalog # 90075) and anti-mouse IgG2b produced in goat as the secondary antibody (Sigma, catalog #A3562). The membrane was developed by incubation in AP development solution (0.015% BCIP, 0.03% NBT in 0.5 M Tris-HCl, pH 9.5) for 2 min and then the picture was taken.

### Database search

The MS/MS data were processed using Maxquant search engine (v.1.5.2.8). Tandem mass spectra were searched against *UniprotKB* and *B. napus* database (http://www.genoscope.cns.fr/brassicanapus/) concatenated with a reverse decoy database. A maximum of two missed cleavages were allowed for trypsin. Carbamidomethyl on cysteine was specified as fixed modification and sulfenylation on “Met” were specified as variable modifications. Following the procedures, we set “First search” as an initial andromeda search, after the recalibration, “Main search” was performed. The mass tolerance for precursor ions was set as 20 ppm in “First search” and 5 ppm in “Main search”, and the mass tolerance for fragment ions was set as 0.02 Da. The identifications from the “First search” are not used in the actual results, and the false discovery rate (FDR) only been estimated after “Main search”. FDR was adjusted to < 1% and minimum score for modified peptides was set > 40. For quantification method, iodoTMT-6plex was selected. All the other parameters in MaxQuant were set to default values. The site localization probability was set as > 0.75.

### Subcellular localization prediction and GO enrichment analysis

The subcellular localization of proteins was predicted by soft Wolfpsort (http://www.genscript.com/psort/wolf_psort.html), which is an updated version of PSORT/PSORT II for the prediction of eukaryotic sequences. For further understanding of redox network, GO enrichment analysis was performed to predict the functional characteristics of identified proteins. For GO enrichment analysis, proteins were classified by GO annotation into three categories: biological process, cellular compartment and molecular function. For each category, a two-tailed Fisher’s exact test was employed to test the enrichment of the differentially expressed protein against all identified proteins. A *p*-value < 0.05 was considered statistically significant. Proteins in *B. napus* were aligned to all *Arabidopsis* proteins by BLASTP (Jacob et al., 2008), and the most significant proteins in *Arabidopsis* based on a probability threshold of 10^-5^ were selected for enrichment analysis.

### Recombinant protein expression and purification

RNA was extracted from *B. napus* leaves using Transzol reagent (TransGen, ET101) according to manufacturer’s instructions. cDNA was reverse-transcribed using TransScript One-Step gDNA Removal and cDNA Synthesis SuperMix (TransGen, AE311) according to the manufacturer’s instructions. *BnA09.PK* (BnaA09G0493100ZS) and *BnA02.MDH2* (BnaA02G0032600ZS) were cloned into PET28a-c (+) expression vector using primers (PK-3100ZS-F: 5´GCGGAATTCATGCATTCGAGCCATCTCC3´, PK-3100ZS-R: 5´GCGCGGCCGCATCCTCAAGCTCGATGATCTTGA3´; MDH2-2600ZS -F: 5´ATGTCCGTGGAGATGCC3´, MDH2-2600ZS-R: 5´TTTCCTGATGAAGTCAACACCTTTCT3´). The constructs were expressed in *E. coli* cells (BL21DE3 pLySs). For expression induction, isopropyl ß-D-thiogalactoside (IPTG) was added to the culture to induce the expression of target proteins at a final concentration of 0.3 mM and bacteria were grown with agitation (200 rpm) for 8 h at 25°C. Proteins were extracted using a buffer containing 50 mM NaH_2_PO_4_, 300 mM NaCl, 10 mM imidazole and 1 mM phenyl-methyl-sulphonyl-fluoride (PMSF), adjusted to pH 8.0 with NaOH. To purify soluble (6xHis) recombinant proteins, the supernatants were incubated with Ni-NTA resin (Sangon Biotech). Elution of the bound proteins from the column was achieved with 50 mM NaH_2_PO_4_, 300 mM NaCl and 250 mM imidazole, adjusted to pH 8.0 with NaOH.

### Enzyme activity assay

Pyruvate kinase (PK) catalyzes the production of pyruvate from phosphoenolpyruvate (PEP). Pyruvate kinase activity was detected by coupling the production of pyruvate to the conversion of NADH to NAD^+^ by lactate dehydrogenase (Zhang and Liu, 2016). Reactions were started by the addition of purified BnA09.PK, and the linear region of the reaction lasts at least 5 min at 25°C. Standard reaction mixtures contained 100 mM HEPES-KOH, pH 7.0, 5% polyethylene glycol (PEG) 8000, 10 mM KCl, 12 mM MgCl_2_, 2 mM PEP, 1 mM ADP, 0.2 mM NADH, and 1.5 units mL^-1^ desalted rabbit muscle lactate dehydrogenase. MDH catalyzes the conversion between oxaloacetic acid (OAA) and L-malate. The reaction mixtures contained 100 mM Tris-HCl (pH 7.4), 0.2 mM NADH and 2 mM OAA (Mekhalfi et al., 2014). After adding 1 μg BnA02.MDH2 protein, the reaction was started. For H_2_O_2_ treatment in the enzyme assay, the final H_2_O_2_ concentration in reaction buffer was 0.2 mM, 0.5 mM, 1 mM, 2 mM, 5 mM, 10 mM. The standard reaction system was taken as control. Enzyme activity was analyzed assayed by recording the changes in absorbance of NADH at the wavelength of 340 nm using an ultraviolet spectrophotometer.

### Metabolite analyses by LC-MS/MS

Metabolite extraction and analyses was performed using a modified method described previously (Luo et al., 2007; Guo et al., 2014). The sampling time of plant materials for metabolite analysis was the same as that for proteomic analysis. About 100-200 mg of leaf was harvested and ground in liquid nitrogen. 1.8 mL of methanol/chloroform (7:3, v/v; -20°C) was added and mixed by vortex followed by the addition of 0.8 μg PIPES as the internal standard. The mixtures were maintained for 2 h at -20°C with occasional vortexing. Polar metabolites were extracted from the methanol/chloroform phase by the addition of 1.6 mL water to each sample and then centrifuged at 1000 rpm after vigorous vortexing. The methanol-water phase was then transferred to a new tube, and another 1.6 mL of water was added to each sample to extract polar metabolites one more time. The extracts were dried by N_2_ aspiration at 50°C. The dried extract was dissolved with 200 μL water and filtered with 0.45 mM cellulose acetate centrifuge filters for metabolite analyses by LC-MS/MS. The metabolites were quantified by comparing with the peak area of internal standard.

## Results

### IodoTMT-based proteomic approach for identification of redox-sensitive proteins in *B. napus*

**Figure 1:**
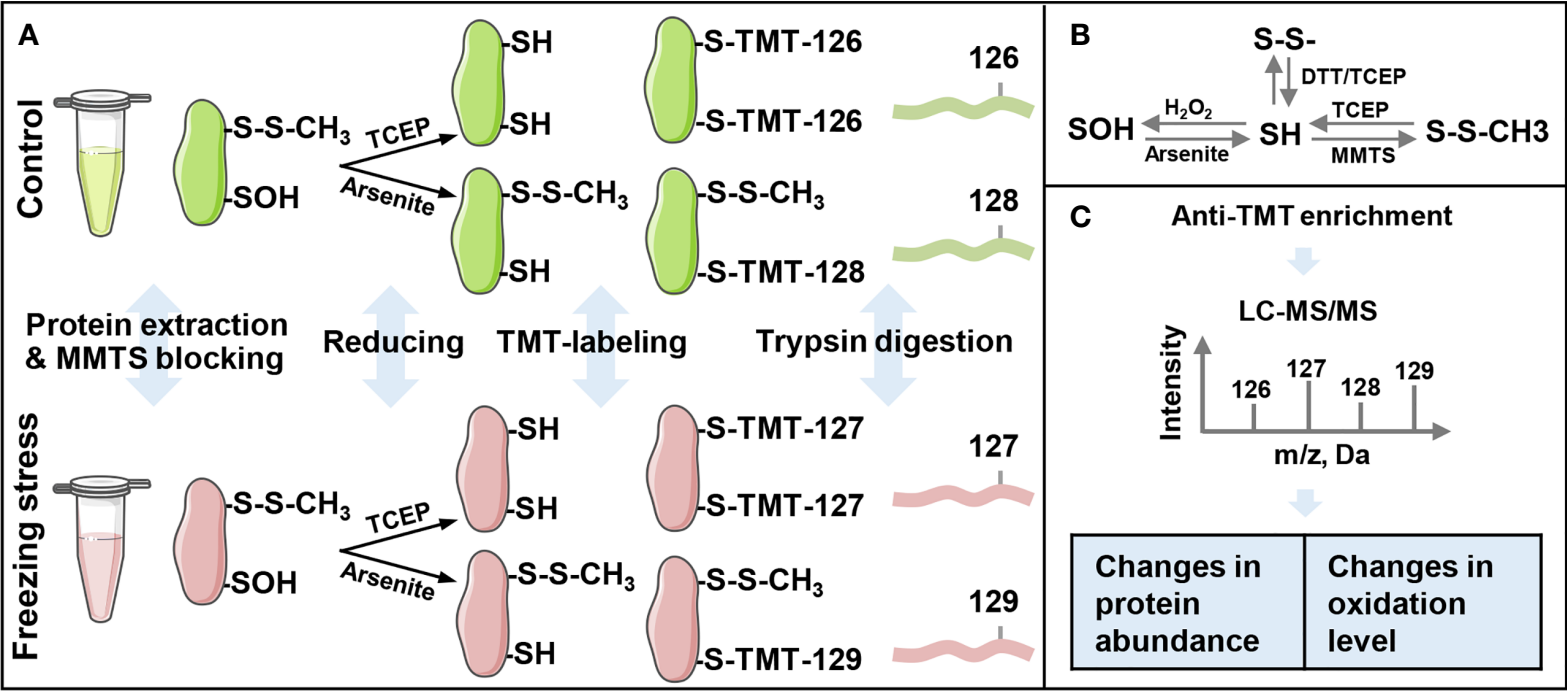
Workflow of the iodoacetyl tandem mass tags (iodoTMT)-based proteomic approach for the identification of sulfenylated cysteine sites. **(A)** The schematic of protein extraction, labeling and trypsin digestion processes. The iodo-TMT labels TMT-126 and TMT-127 were used to label free thiol (R-SH), and labels TMT-128 and TMT-129 were used to label sulfenylated thiol (R-SOH). **(B)** Thiol (SH) redox state change under different oxidants and reducing agents. SH, sulfhydryl group; S-S, disulfide bond; SOH, sulfenylation group; MMTS, S-Methyl Methanethiosulfonate; TCEP, Tris (2-carboxyethyl) phosphine; DTT, dithiothreitol. **(C)** Anti-TMT™ antibody-based enrichment and LC-MS/MS analysis of peptides’ intensity. Changes in expression levels are calculated by the ratio of TMT-127 (freezing stress) to TMT-126 (control) and changes in sulfenylation levels are calculated by the ratio of %R-SOH (TMT-129/TMT-127) under freezing stress to %R-SOH (TMT-128/TMT-126) under control condition.

**Figure 2:**
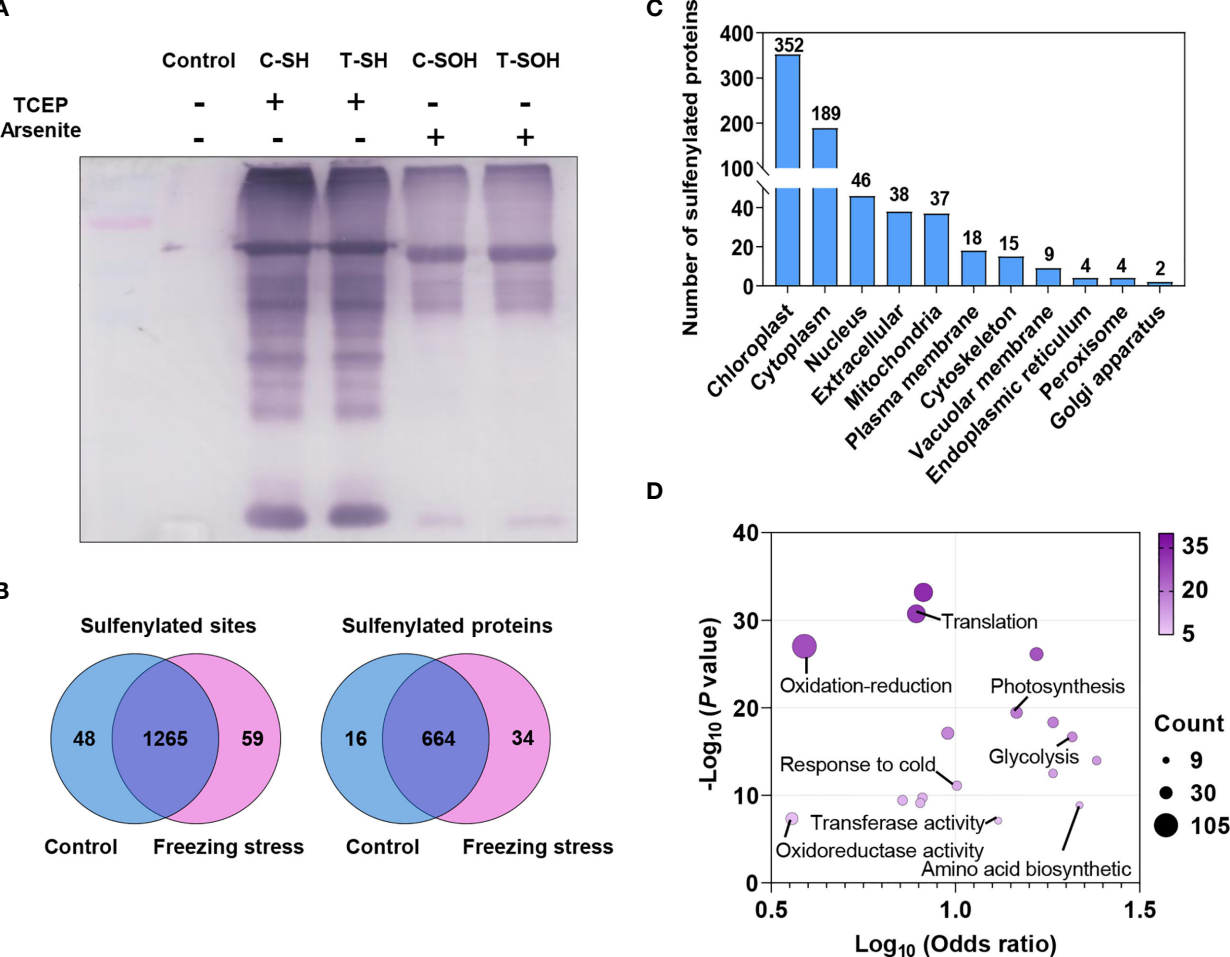
Sulfenylated sites and proteins identified by proteomic analysis in *B napus*. **(A)** The detection of total proteins reduced by TCEP and arsenite was performed by Western blotting using anti-TMT™ antibody. Reduced (SH) and sulfenylated (SOH) proteins in control **(C)** and freezing treatment after cold acclimation (T) samples were labeled, respectively. Proteins without label were used as control. **(B)** Venn diagram showing the shared sulfenylated sites and proteins identified in control and stressed samples. The number of sites and proteins are listed in each diagram component, respectively. **(C)** The distribution of predicted subcellular localizations of sulfenylated proteins. **(D)** GO enrichment analysis of sulfenylated proteins. The Log_10_ (Odds ratio) is plotted on the x-axis. The size and color of dots indicate the number of sulfenylated proteins and the degree of enrichment (-Log_10_ (*P* value), *P* < 0.05), respectively.

An iodoTMT-based proteomic approach was used in this study to investigate the change in protein abundance and sulfenylation level under freezing stress after cold acclimation in *B. napus* (**Figure 1**). After cold acclimation, the leaves of *B. napus* displayed a low H_2_O_2_ content before freezing treatment (0 h), showing a similar level of H_2_O_2_ compared to that under normal condition (CK). However, an obvious accumulation of H_2_O_2_ was observed after freezing treatment, especially after 3-hour treatment (**Figure S1**). In order to obtain as many modified proteins as possible, we set the sampling time at 4 hours after treatment to perform the proteomic identification. Blocking the sulfhydryl group (-SH) on cysteine residues by MMTS to minimize the thiol (R-SH) from oxidation during protein extraction, which is necessary for maintaining the initial redox state of proteins (**Figures 1A, B**). After that, samples were split in two to perform the reduction process of TCEP and arsenite, respectively. Anti-TMT™ antibody was used to enrich the peptides followed by the digestion of labeled proteins (**Figures 1A, C**). Then the enriched peptides were identified and quantified by LC-MS/MS (**Figure 1C**). Finally, the change in protein abundance and sulfenylation level can be analyzed. Western blot result showed a weak reduction ability of arsenite compared with TCEP (**Figure 2A**). The Pearson coefficient between replicates (**Figure S2A**) and quality control of MS (**Figure S2B**) proved a reliable quantitative proteomic data.

A total of 968 unique sulfenylated peptides were obtained from the MS data (**Table S1** **)** matched to 1,372 sulfenylated sites in 714 proteins **(** **Table S2** **)**. Among these sulfenylated sites and proteins, 1,265 sites (92.2%) in 664 proteins existed in both control and freezing treated samples (**Figure 2B**). The majority of these sulfenylated proteins were predicted to localize in chloroplast, cytoplasm, nucleus, extracellular and mitochondria, and a few proteins were predicted to localize in plasma membrane, cytoskeleton, vacuolar membrane, endoplasmic reticulum, peroxisome and golgi apparatus (**Figure 2C**). These sulfenylated proteins were notably enriched in glycolysis, tricarboxylic acid cycle and photosynthesis processes and they were more likely to function in chlorophyll binding, transferase activity and GTPase activity (**Figure 2D**). Taken together, these findings demonstrate that the iodoTMT-based proteomic approach can effectively identify a large number of *in vivo* sulfenylated proteins.

### Freezing stress caused a changed abundance of proteins *in vivo*

**Figure 3:**
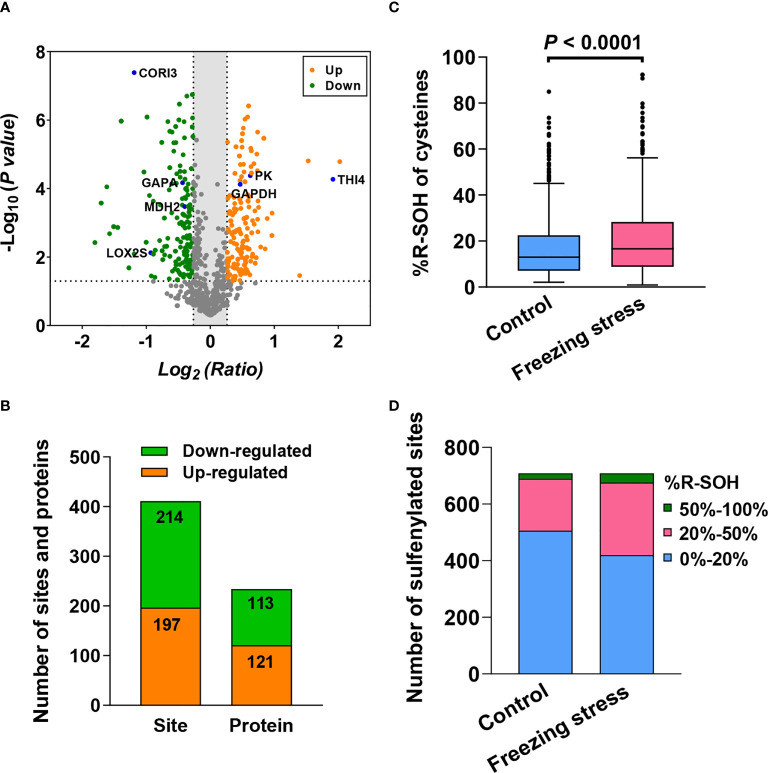
Freezing stress after cold acclimation caused the change in protein abundance and sulfenylation level (%R-SOH) of sites and proteins in *B napus.* **(A)** Volcano plot of the 857 sites quantified in at least 2 out of 3 replicates under both control and stressed conditions. The statistical significance -Log_10_ (*P* value) on the y-axis and the expression level change Log_2_ (Ratio) on the x-axis. Ratio means the ratio of cysteine level under stress to that under control condition. Red dots represent the sites with up-regulated expression level (ratio > 1.2) and green dots represent the sites with down-regulated expression level (ratio < 0.83). The dotted line on the y-axis shows *P* value of 0.05, and the dotted lines on the x-axis show ratio of 1.2 and 0.83. Some known proteins in *A thaliana* are marked in blue in the volcano plot. **(B)** The number of sites and proteins with significant abundance changes under stress (*P* < 0.05). **(C)** Sulfenylation level of the 709 sites quantified in at least 2 out of 3 replicates under control and stressed conditions. %R-SOH means the proportion of modified state abundance to the total abundance of a single site, which was used to indicate the sulfenylation level. **(D)** %R-SOH distribution of the 709 sites under control and stressed conditions.

In order to analyze the change in protein abundance under freezing stress after cold acclimation in *B. napus*, 857 labeled R-SH sites in 458 proteins identified in at least two replicates were analyzed (**Table S3**). By labelling the total cysteines (R-SH) with TMT-126 (Control) and TMT-127 (Stress), we can evaluate the abundance change of sites and proteins by calculating the normalized intensity ratio of R-SH under stress condition to that under control condition. Among these 857 sites, there were 411 sites in 233 proteins showed significant changes in their abundance under freezing stress in *B. napus* (**Figure 3A** and **Table S4**). The abundance of 197 sites in 121 proteins were up-regulated and 214 sites in 113 proteins were down-regulated under freezing stress after cold acclimation (**Figure 3B** and **Table S4**). GO enrichment analysis of these differentially expressed proteins (DEPs) showed that the up-regulated proteins were enriched in the processes of nucleotides synthesis and metabolism, translation and translation elongation and activities of nucleoside diphosphate kinase and translation elongation factor (**Figure S3A**). The down-regulated proteins were involved in carbon metabolism process and molecular functions such as ribulose-bisphosphate carboxylase activity, chlorophyll binding and lipid transport (**Figure S3B**).

### Freezing stress caused fluctuations in the landscape of protein sulfenylation

In order to explore the effect of freezing stress after cold acclimation on the overall sulfenylation level of proteins in *B. napus*, we analyzed the %R-SOH of sulfenylated sites to reflect their sulfenylation level under control (%R-SOH = TMT-128/TMT-126) and stressed conditions (%R-SOH = TMT-129/TMT-127). There were 709 R-SOH sites in 402 proteins were identified in at last two replicates under both conditions (**Table S5**). By calculating the ratio of %R-SOH under stressed condition to that under control condition, we can estimate the sample-specific ratio of sulfenylated level to expression level of cysteines under stress (**Table S5**). In general, the treated sample displayed an increased %R-SOH when compared with the control samples (**Figure 3C**). This change was mainly due to the transformation from low sulfenylation level (0%-20%) to high sulfenylation level (20%-100%) of sites under freezing stress after cold acclimation (**Figure 3D**). In terms of the predicted subcellular distribution of sulfenylated proteins, the degree of %R-SOH increased in all compartments, especially in chloroplast and cytoplasm (**Figures S4A, B**). These results indicate that freezing stress after cold acclimation widely raise the sulfenylation level of sites and proteins *in vivo*.

**Figure 4:**
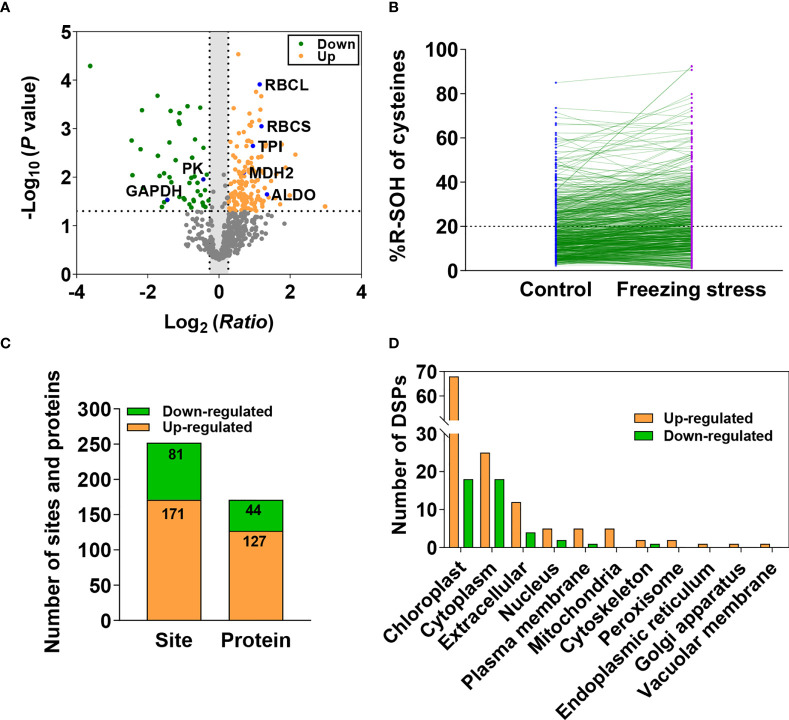
Differentially sulfenylated sites and proteins identified under freezing stress after cold acclimation in *B napus*. **(A)** Volcano plot of the 709 sites showing H_2_O_2_-dependent sulfenylation level change (ratio of %R-SOH) of cysteines, statistical significance -Log_10_ (*P* value) on the y-axis and sulfenylation level change Log_2_ (*Ratio*) on the x-axis. Ratio means the ratio of %R-SOH under fstress condition to %R-SOH under control condition. Red dots represent the sites with increased sulfenylation level and green dots represent the sites with decreased sulfenylation level (*P* < 0.05). The dotted line on the y-axis shows *P* value of 0.05, and the dotted lines on the x-axis show ratio of 1.2 and 0.83. Sulfenylated proteins identified previously in *A thaliana* are marked in blue in the volcano plot. **(B)** The %R-SOH of the 709 sites under control (blue) and stressed (red) conditions. The %R-SOH is plotted on the y-axis and the split line represents %R-SOH equal to 20%. **(C)** The number of differentially sulfenylated sites and proteins (*P* < 0.05). **(D)** Subcellular localizations of the differentially sulfenylated proteins (DSPs).

Differentially sulfenylated sites and proteins under freezing stress after cold acclimation were identified by the threshold of the %R-SOH ratio described above. The sites increased (ratio > 1.2) and decreased (ratio < 0.83) at their sulfenylation level were screened, respectively. A total of 714 sulfenylated proteins can be quantitatively evaluated, some of which have been reported as sulfenylated proteins in *A. thaliana* such as ribulose 1,5-bisphosphate carboxylase/oxygenase large subunit (RBCL) and glyceraldehyde-3-phosphate dehydrogenase (GAPDH) (**Figure 4A**; **Table S2**) (Alvarez et al., 2009; Wang et al., 2012; Liu et al., 2014; Liu et al., 2015). When comparing the %R-SOH of a single site under control and stressed conditions, besides a large number of sites with an increased sulfenylation level, a considerable proportion of sites displayed a decreased sulfenylation level, including some highly sulfenylated sites (**Figure 4B**). Among them, 252 sites in 171 proteins were differentially sulfenylated (**Figure 4C**; **Table S6**). 171 sites in 127 proteins were increased and 81 sites in 44 proteins were decreased in %R-SOH (**Figure 4C**; **Table S6**). There were 34 highly sulfenylated sites in 32 proteins with an increasing sulfenylation level (**Table S7**). Interestingly, 53 differentially sulfenylated sites in 49 proteins changed from low to high sulfenylation level and some representative differentially sulfenylated sites and proteins were listed in **Table 1**. Moreover, 70 proteins displayed an increase sulfenylation level with a decreased expression level (**Figure S5A**). 12 proteins showed an increased trend both in expression level and sulfenylation level, while 11 proteins showed decreased trend both in expression level and sulfenylation level (**Figure S5A**). Most of the differentially sulfenylated proteins (DSPs) were predicted to localize in chloroplast and cytoplasm (**Figure 4D**). Based on GO enrichment analysis, proteins with an increased sulfenylation level were enriched in carbon utilization, photosynthesis and glycolysis, and molecular functions such as aldolase activity, catalase activity and chlorophyll binding (**Figure S5B**). These results suggest that the sulfenylation level of proteins are rearranged under freezing stress after cold acclimation, and stress-induced sulfenylation notably happen upon primary metabolism and enzymes in chloroplasts and cytoplasm.

**Table 1:** **Differentially sulfenylated sites and proteins changed from low to high sulfenylation level**.

Position	Gene name	%R-SOH-CK	%R-SOH-T	Ratio	p-value	Protein description
350	BnaAnng23670D	0.186	0.286	1.538	0.010	ATPase 2, plasma membrane-type-like
116	BnaC08g32210D	0.189	0.296	1.567	0.012	Calvin cycle protein CP12-2, chloroplastic
215	BnaC06g28360D	0.177	0.277	1.568	0.008	Probable lactoylglutathione lyase, chloroplast
129	BnaA09g22540D	0.183	0.288	1.575	0.018	Chlorophyll a-b binding protein CP26, chloroplastic-like
284	BnaA02g00100D	0.132	0.216	1.629	0.008	Malate dehydrogenase 2, glyoxysomal
56	BnaC08g45680D	0.146	0.238	1.630	0.026	Coproporphyrinogen-III oxidase 1, chloroplastic
145	BnaA06g05150D	0.186	0.303	1.631	0.048	Superoxide dismutase 1
440	BnaCnng36310D	0.174	0.284	1.636	0.010	Fanconi anemia group I protein-like
223	BnaA03g53180D	0.149	0.248	1.663	0.006	Catalase-2
56	BnaA02g00470D	0.149	0.251	1.680	0.043	40S ribosomal protein S6-2-like
464	BnaA05g05840D	0.131	0.222	1.692	0.031	Ribulose bisphosphate carboxylase/oxygenase activase
227	BnaC08g36130D	0.178	0.302	1.697	0.025	Protein THYLAKOID FORMATION 1, chloroplastic-like
311	BnaCnng09500D	0.129	0.220	1.704	0.027	Haloacid dehalogenase-like hydrolase domain-containing protein
122	BnaA08g07940D	0.125	0.218	1.737	0.002	Photosystem I reaction center subunit III,
55	BnaA10g18270D	0.120	0.212	1.755	0.005	COX assembly mitochondrial protein 2-like
397	BnaC08g46180D	0.119	0.211	1.775	0.044	Glyceraldehyde-3-phosphate dehydrogenase GAPB
522	BnaA02g03770D	0.196	0.349	1.784	0.002	—
13	BnaC02g07040D	0.182	0.328	1.801	0.008	Nitrilase 2-like
573	BnaA05g00900D	0.146	0.264	1.813	0.001	Photosystem I P700 apoprotein A1 (chloroplast)
375	BnaC08g04910D	0.173	0.325	1.884	0.035	Triacylglycerol lipase-like 1
208	BnaC06g28580D	0.130	0.246	1.889	0.004	Cysteine/Histidine-rich C1 domain family protein
141	BnaA03g24350D	0.120	0.226	1.891	0.029	—
331	BnaA09g17390D	0.139	0.265	1.904	0.001	2-oxoglutarate dehydrogenase, mitochondrial-like
213	BnaA03g38340D	0.115	0.219	1.904	0.006	—
316	BnaC04g20620D	0.164	0.319	1.954	0.015	50S ribosomal protein L1, chloroplastic-like
77	BnaAnng27610D	0.116	0.235	2.019	0.012	Non-specific lipid-transfer protein D-like
149, 151	BnaC05g06200D	0.122	0.255	2.094	0.029	Violaxanthin de-epoxidase, chloroplastic-like
56	BnaA06g17780D	0.133	0.283	2.127	0.011	Tubulin beta-2 chain
127, 132	BnaC03g65520D	0.146	0.313	2.141	0.025	Aquaporin PIP2-7-like
227	BnaCnng05580D	0.096	0.212	2.198	0.001	Beta carbonic anhydrase 1, chloroplastic-like
140	BnaA03g25540D	0.105	0.236	2.236	0.029	ATP synthase gamma chain 1, chloroplastic
233	BnaA09g52790D	0.113	0.259	2.283	0.004	Myrosinase-like
230	BnaC05g42240D	0.095	0.222	2.323	0.035	Superoxide dismutase 1, mitochondrial-like
179	BnaC07g15490D	0.087	0.216	2.493	0.002	V-type proton ATPase subunit B1-like
117	BnaA06g30160D	0.099	0.302	3.052	0.009	Cysteine-rich repeat secretory protein 55-like
304	BnaA04g07450D	0.072	0.264	3.664	0.006	Glutamine synthetase, chloroplastic
468, 469, 473	BnaA01g18450D	0.040	0.317	7.894	0.040	Pyruvate, phosphate dikinase 1, chloroplastic

CK, the control samples; T, the treated samples.

In our previous studies, the change in protein abundance and sulfenylation level under salt-stressed condition was investigated in *B. napus* by the same approach (Yu et al., 2021). Comparison showed that 720 (74.4%) sulfenylated peptides were overlapped between the two studies (**Figure S6A**). Among the proteins with up-regulated expression level, 11 proteins were up-regulated under both stressed conditions (**Figure S6B**). In addition, 16 proteins with increased sulfenylation level and 11 proteins with decreased sulfenylation level were identified under both freezing and salt stressed conditions (**Figures S6C, D**). These results indicate the stability of the iodoTMT-based approach for identification of sulfenylated proteins and freezing and salt stresses induce common and unique sulfenylation of proteins.

### Freezing-induced alteration in primary metabolism pathway

**Figure 5:**
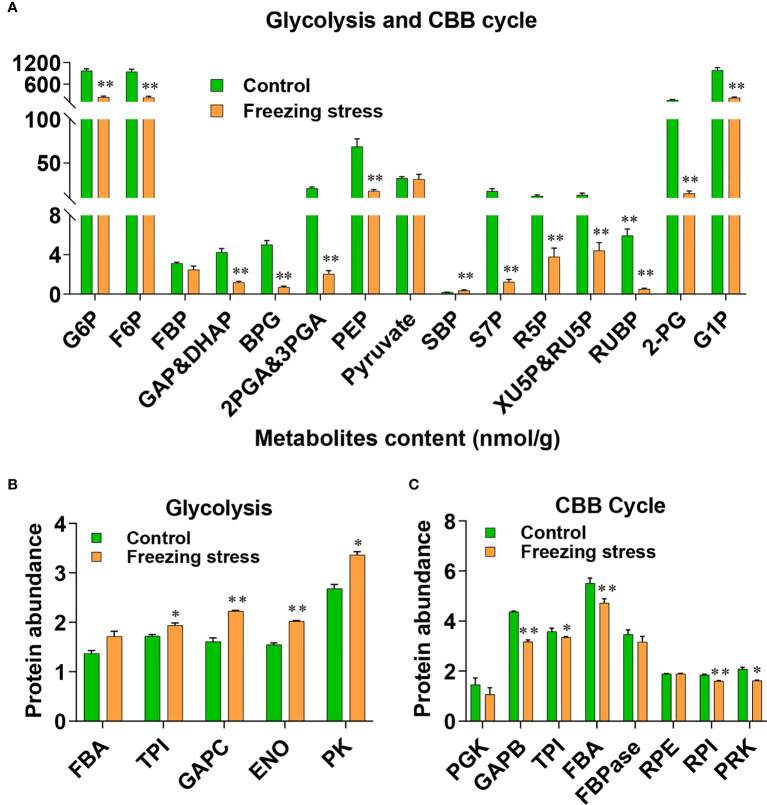
Metabolite level and protein abundance analysis of glycolysis pathway and Calvin-Benson (CBB) cycle. **(A)** Metabolite analysis of glycolysis pathway and CBB cycle. G6P, glucose-6-phosphate; F6P, fructose-6-phosphate; FBP, fructose-1,6-bisphosphate; GAP, glyceraldehydes-3-phosphate; DHAP, dihydroxyacetone-phosphate; BPG, 1,3-biophosphoglycerate; 2PGA, 2-phosphoglycerate; 3PGA, 3-phosphoglycerate; PEP, phosphoenol pyruvate; SBP, sedoheptulose-1,7-bisphosphate; S7P, sedoheptulose-7-phosphate; R5P, ribose-5-phosphate; RU5P, ribulose-5-phosphate; XU5P, xylulose 5-phosphate; RUBP, ribulose-1,5-bisphosphate; 2-PG, 2-phosphoglycolate; G1P, glucose-1-phosphate. Values are means ± SE (n = 6 biological replicates, *P < 0.05, **P < 0.01 compared with control using Student’s t-test). **(B, C)** Protein abundance of enzymes involved in glycolysis pathway **(B)** and CBB cycle **(C)**. FBA, Fructose-1,6-bisphosphate aldolase; TPI, Triose phosphate isomerase; GAPC, glyceraldehyde-3-phosphate dehydrogenase; ENO, enolase; PK, pyruvate kinase; PGK, phosphoglycerate kinase; GAPB, glyceraldehyde-3-phosphate dehydrogenase B; FBPase, fructose-1,6-biphosphatase; RPE, ribulose-5-phosphate-3-epimerase; RPI, ribose 5-phosphate isomerase; Values are means ± SE (n = 3 biological replicates, *P < 0.05, **P < 0.01 compared with control using Student’s t-test).

Some DSPs identified by our proteomic data as well as a large number of the known redox-sensitive proteins participate in several pathways of primary metabolism, including glycolysis, the Calvin-Benson-Bassham (CBB) cycle, and the tricarboxylic acid (TCA) cycle (**Figure S7**). The results from metabolite profiling analysis also showed decreased level of metabolites in glycolysis and CBB cycle pathways (**Figure 5A**). Based on the analysis of total cysteine level in proteins, we obtained the protein abundance of these enzymes. The glycolytic enzymes including triose phosphate isomerase (TPI), glyceraldehyde-3-phosphate dehydrogenase C (GAPC), enolase (ENO) and pyruvate kinase (PK) showed an increased abundance under freezing stress after cold acclimation (**Figure 5B**; **Table S8**), while enzymes like glyceraldehyde-3-phosphate dehydrogenase B (GAPB), TPI, fructose-1,6-bisphosphate aldolase (FBA) and ribose 5-phosphate isomerase (RPI) in CBB cycle were decreased in their abundance under stressed condition (**Figure 5C**; **Table S8**). However, the results from transcriptome analysis showed that key enzymes of glycolysis such as FBA, TPI, GAPC and PK did not decrease but significantly increase at the transcriptional level (**Figure S8A**'; **Table S9**). Similarly, the gene expression level of enzymes involved in the CBB cycle also showed no tendency to decrease, except for PGK and FBA (**Figure S8B**; **Table S9** **)**. No significant difference in metabolite level was observed in TCA cycle (**Figure S8C**). In addition, the reductive power, the ratio of NADPH/NADP^+^ in cells was significantly up regulated (**Figure S8D**). These results suggest that posttranslational modification, in addition to transcriptional and translational changes, plays an important role in metabolic response to freezing stress after cold acclimation.

### Activity of pyruvate kinase and malate dehydrogenase was inhibited by H_2_O_2_

**Figure 6:**
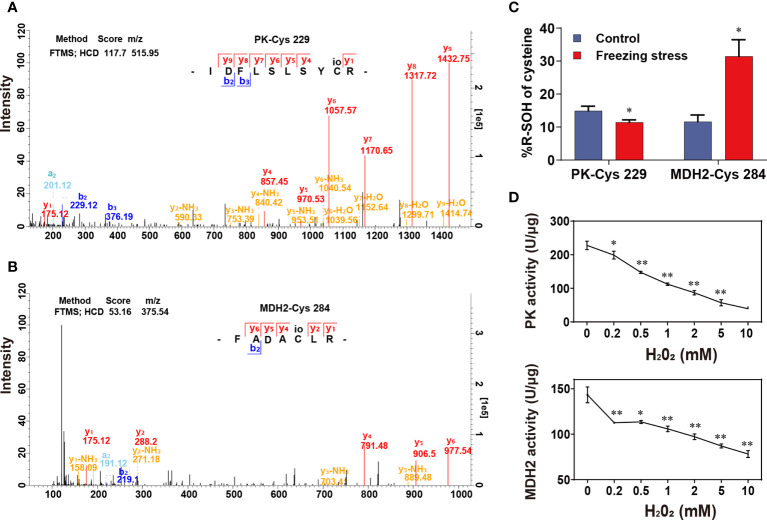
Effect of H_2_O_2_-induced sulfenylation on enzyme activity of pyruvate kinase (PK) and malate dehydrogenase 2 (MDH2). **(A, B)** Mass spectra of peptides containing PK-Cys 229 **(A)** and MDH2-Cys 284 **(B)**. The mass spectra scanning using fourier transform mass spectrometry (FTMS) and the fragmentation method is higher energy collision induced dissociation (HCD); Score, match degree between the fragment produced by the mass spectra and that recorded in the database; m/z, the ratio of mass to charge of the peptide; “IDFLSLSYCR” and “FADACLR” were the peptides sequence of PK and MDH2 identified in this study. **(C)** %R-SOH change of PK and MDH2. Values are means ± SD (n = 3 biological replicates, *P < 0.05 compared with control using Student’s t-test). **(D)** Enzyme activity of PK and MDH2 under H_2_O_2_ treatment *in vitro*. Values are means ± SD (n = 3 technical replicates, *P < 0.05, **P < 0.01 compared with control using Student’s t-test).

**Figure 7:**
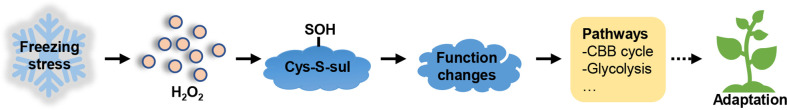
Schematic representation of the metabolic response induced by freezing stress. Under freezing stress, H2O2 accumulation induces sulfenylation of proteins, consequently affecting their functions, such as enzyme activity and binding ability. The modified proteins are involved in several metabolic processes, including CBB cycle and glycolysis, which further help the plants adapt to freezing stress.

GO enrichment analysis revealed that a great number of enzymes were differentially sulfenylated (**Figure S5B**). Therefore, we explore the regulation of sulfenylation in enzyme activity. Cytosolic pyruvate kinase (PK) and cytosolic malate dehydrogenase (MDH2) were two key enzymes in glycolysis and TCA cycle, respectively. Both PK and MDH2 were differentially sulfenylated under freezing stress after cold acclimation in our data (**Table S6**). Only one peptide containing the sulfenylated site of cysteine 229 was identified in PK from the MS data (**Figure 6A**; **Table S2**), while three peptides containing the sulfenylated site of cysteine 147, 284 and 297, respectively, were identified in MDH2 (**Figure 6B**; **Table S2**). Both PK-Cys 229 and MDH2-Cys 284 showed significant changes in %R-SOH level (**Table S6**). The %R-SOH of PK-Cys 229 was decreased, whereas that of MDH2-Cys 284 was increased under stress (**Figure 6C**). We successfully purified these two proteins from *E*. *coli* (**Figures S9A, B**), and the enzyme activity assay under H_2_O_2_ treatment *in vitro* were performed. We observed a significant reduction in activity of PK and MDH2 after adding H_2_O_2_ in the reaction system and the degree of inhibition was enhanced with the increase of H_2_O_2_ concentration (**Figure 6D**). These results indicate that H_2_O_2_-induced inhibition of PK or MDH2 activity might be due to redox modification such as sulfenylation. A schematic model was drawn according to our results, showing the response and adaptation of *B. napus* plants under freezing stress (**Figure 7**).

## Discussion

Various methods such as saturation differential in-gel electrophoresis (DIGE) and isotope-coded affinity tagging (ICAT) have been used to identify PTMs in plants (Leichert et al., 2008; Go et al., 2010; Zhu et al., 2014). But the number of proteins identified by previous methods is very limited and the reproducibility between different methods and repetitions is poor. IodoTMT-based strategy provides a robust and reproducible method for identification of the sulfenylated sites and quantification of the relative abundance of R-SH and R-SOH in proteins. Many 6-plex tandem mass tag (TMT) reagents have been used to quantify sulfenylation, and were assessed to reduce run-to-run variance (Murray et al., 2012; Pan et al., 2014). Due to the small amplitude change of modification, accurate determination of the endogenous levels of thiol modification have been proved to be difficult. Combined anti-TMT enrichment and LC-MS/MS analysis can largely improve the sensitivity of measurement (Wojdyla et al., 2015). We thus chose this method to screening differentially expressed and differentially sulfenylated proteins. In addition, little knowledge has been obtained on identification of endogenous sulfenylated proteins under abiotic stresses in plants. In our study, we focus on the DSPs induced by freezing stress after cold acclimation, which can cause severe damage to overwintering plants such as *B. napu*s. Finally, we obtained 252 differentially sulfenylated sites in 171 proteins (**Table S6**), and the putative localization and function of these proteins were analyzed, which has an important reference significance for redox regulation of plant adaptability to stresses. Compared with the result on identification of sulfenylated cysteines under salt stress using the same method in our previous study (Yu et al., 2021), 74.4% peptides were found to be overlapped under both salt and freezing stress. However, only a few differentially expressed and differentially sulfenylated proteins were overlapped under the two stresses (**Figures S6C, D**). This indicated that the iodoTMT-based strategy has a good repeatability in comparing the modification levels between different samples. Besides, different stress treatments may induce the modification of different proteins.

Freezing stress after cold acclimation caused a decreased metabolic level of glycolysis and CBB cycle (**Figure 5A**). Redox regulation by S-nitrosylation (SNO) and S-glutathionylation (SSG) is required for plant adaptation to biotic and abiotic stresses, allowing fine-tuning of the CBB cycle (Michelet et al., 2013). In combination with metabolic, protein abundance and transcriptional analyses, we found strong inconsistencies in alteration of mRNA level, protein abundance and metabolic changes under stress (**Figure 5** and **Figure S8**). For example, though the content of metabolites S7P, Xu5P, Ru5P and RuBP involved in the CBB cycle was significantly reduced (**Figure 5A**), there was no significant difference in mRNA level and protein abundance of TPI and FBPase under stress (**Figure 5C** and **Figure S8B**). Interestingly, TPI, FBA and FBPase, which catalyze the upstream reactions of these metabolites, was significantly increased at their sulfenylation level under stress, which may explain metabolic changes to some extent (**Figure 5A**, **Table S6**). PRK is an essential enzyme of the CBB cycle to produce RuBP in photosynthesis, and GAPDH/CP12/PRK complex plays a pivotal in the regulation of the CBB cycle (Marri et al., 2005). The ATP site and Ru5P site can be disrupted by the disulfide bond formed between Cys 17 and Cys 56 after oxidization, this redox-controlled switch further regulates the CBB cycle through the activation/deactivation of PRK and the reversible formation of the GAPDH/CP12/PRK ternary complex (Marri et al., 2009; Yu et al., 2020). We found that the sulfenylation level of PRK was significantly increased (**Figure S7** and **Table S6**) and the content of its product RuBP was significantly decreased under stress (**Figure 5A**). The mRNA level of *PRK* was not significantly altered (**Figure S8B**), while the protein abundance of PRK showed a significant decrease under stressed condition (**Figure 5C**). These results suggest that post-transcriptional regulation and PTMs may work together in the regulation of PRK function in response to freezing stress after cold acclimation.

Many chloroplastic isoforms of glycolytic enzymes involved in the CBB cycle, such as FBA, TPI, PGK, PK and so on, were all identified as sulfenylated proteins in *B. napus* (**Figure S7**). Cytosolic glycolytic enzymes including GAPC, PGK, PGAM and ENO have been identified as potential targets of redox modifications in various studies in *A. thaliana* (Dumont and Rivoal, 2019). The sulfenylated level of GAPC and PK were significantly decreased (**Table S6** and **Figure S7**), and the mRNA level and protein abundance of TPI, GAPC, and PK were increased (**Figure 5B**). Moreover, the content of metabolites BPG, 2PGA, 3PGA and PEP in the glycolytic pathway was significantly reduced under stress (**Figure 5A**). H_2_O_2_ can repress the *in vitro* activity of cytosolic PK (**Figure 6D**), which was a freezing-induced differentially sulfenylated protein identified by our proteomic approach, consistent with the reduced metabolite contents in glycolysis under freezing stress after cold acclimation (**Figure 5A**). It was also found that the protein abundance of some glycolytic enzymes increased, while that of some Calvin-Benson cycle enzymes decreased under freezing stress after cold acclimation (**Figure S8A**). These results were consistent with a previous study, which revealed that the protein abundance of light-harvesting complex, cytochromes and rubisco in Calvin-Benson cycle was reduced under cold stress (Ermilova, 2020). Plant cells are able to hold a balance between growth and adaptation to stresses, and the reestablishment of the new homeostasis helps plants to have a recovery ability and exhibit a renewed growth by regulating the expression of growth-related genes and stress-related genes (Kazemi-Shahandashti and Maali-Amiri, 2018).

Cysteine is an active site that can be modified by a variety of posttranslational modifications, including oxidative modification such as SOH and SSG, other modification such as acylation, lipidation, acetylation, S-nitrosylation, methylation, palmitoylation and phosphorylation, as well as other active amino acids of the PTMs such as lysine, methionine, arginine and so on (Friso and van Wijk, 2015). It has been found that multiple PTMs such as phosphorylation, monoubiquitination, SSG, SNO control the activity, localization and turnover of these glycolytic enzymes (O'Leary and Plaxton, 2020), indicating that other types of PTMs also play considerable roles in the regulation of these enzymes. Different PTMs and crossover between them have also been extensively identified in eukaryotes (Meyer et al., 2012; Weinert et al., 2013 **;** Zhang et al., 2013). These complex PTMs networks may work together to coordinate metabolic control. Proteomic screens have uncovered widespread SNO and SSG of plant glycolytic enzymes in plant cells, such as FBA, TPI, GAPC, PGK, ENO, PK (Dumont and Rivoal, 2019; O'Leary and Plaxton, 2020). Therefore, the inhibition of metabolic level in glycolysis under freezing stress after cold acclimation might also be regulated by other PTMs. Further study on the mechanism of posttranslational modification network was needed to help us understand the roles of PTMs in regulating plant metabolic adaptation to stresses.

H_2_O_2_ can be generated by electron transport chain during the light reaction in chloroplasts, especially when plants are suffered from environmental changes (Foyer and Noctor, 2016). Apoplast is also a main compartment of oxidative bursts. The accumulated H_2_O_2_ in apoplasts can enter the cytoplasm through water channel proteins called aquaporins in plasma membrane (Rodrigues et al., 2017). Previous studies have reported that the general modification of glycolytic enzymes results in not just slowing down of the metabolism for adaptation to an unfavorable environment, but also promotion of C-flux into the pentose phosphate pathway (PPP) to regenerate NADPH to detoxify ROS (Hildebrandt et al., 2015; Dumont and Rivoal, 2019). Our results also showed the increase in the ratio of NADPH/NADP^+^ under freezing stress after cold acclimation (**Figure S8D**), consistent with the previous findings. Among the glycolytic enzymes, GAPC appears to be one of the most prominent targets of redox modifications, which function as a ‘moonlighting’ protein with redox-dependent changes in subcellular localization and biological function. Its re-localization to the nucleus occurred under sulfenylated conditions displays a moonlighting role as a transcription factor to induce gene expression of antioxidant enzymes (Hildebrandt et al., 2015; Schneider et al., 2018; Dumont and Rivoal, 2019). It remains to be explored whether there are other redox-sensitive proteins that function as moonlighting proteins participating in various actual jobs after oxidization. In future, in-depth functional research and structural analysis of posttranslational modified proteins will be of great significance for understanding of the regulatory function of redox PTMs.

## Data availability statement

The original contributions presented in the study are publicly available in the database iProX (https://www.iprox.cn/) (Ma et al., 2019; Chen et al., 2022). This data can be found here: iProX, PXD034549.

## Author contributions

XY and LG designed this study. LY, ZD and SI performed the experiments. YZ and LY did bioinformatics analysis. LY wrote the manuscript. XY, LG and SL revised the manuscript. All authors contributed to the article and approved the submitted version.

## Funding

This research was funded by the Major Scientific and Technological Projects of Xinjiang Production and Construction Corps of China (2018AA005) and the 111 Project (B20051).

## Acknowledgments

This research was funded by the Major Scientific and Technological Projects of Xinjiang Production and Construction Corps of China (2018AA005) and the 111 Project (B20051). This work is supported by the PTM Biolabs lnc. (Hangzhou, China) for technical assistance Additional supporting information may be found online in the Supporting Information section at the end of the article.

## Conflict of interest

The authors declare that the research was conducted in the absence of any commercial or financial relationships that could be construed as a potential conflict of interest.

## Publisher’s note

All claims expressed in this article are solely those of the authors and do not necessarily represent those of their affiliated organizations, or those of the publisher, the editors and the reviewers. Any product that may be evaluated in this article, or claim that may be made by its manufacturer, is not guaranteed or endorsed by the publisher.
