# Supplementary material for: Proteome-wide identification of S-sulfenylated cysteines reveals metabolic response to freezing stress after cold acclimation in Brassica napus
Source: Front Plant Sci. 2022 Sep 29;13:1014295. doi: 10.3389/fpls.2022.1014295 (PMC9580371; doi:10.3389/fpls.2022.1014295)
Supplement: Supplementary file 2 [file DataSheet_1.pdf]

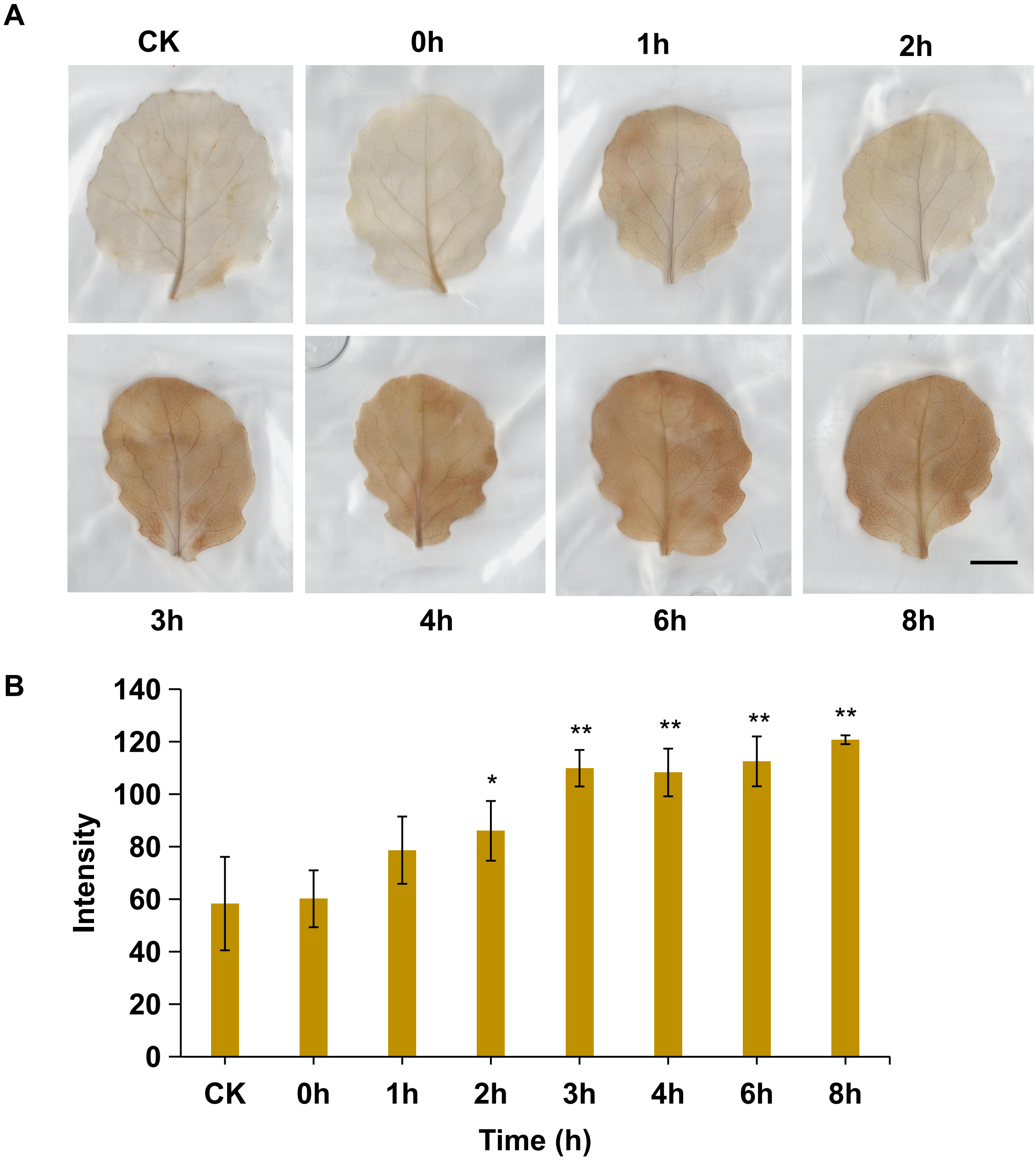

Fig. S1. H_2O_2 level detected by DAB staining.

(A) Leaves of *B. napus* stained by DAB at different time points after freezing treatment. The experiment was repeated three times with three replicates in each group. Bar = 1 cm. CK, leaf from seedling grown under normal condition.

(B) Color depth analysis of DAB stained leaves. The intensity represents mean of gray value which was measured by ImageJ software. Data are means ± SD (n = 3 biological replicates, *, $P < 0.05$, **, $P < 0.01$ relative to control using Student's t-test).

A

Pearson's correlation of *log_2 (ratio)*		Total			Oxidative		
		T1/CK1	T2/CK2	T3/CK3	T1/CK1	T2/CK2	T3/CK3
Total	T1/CK1	1.00	0.93	0.92	0.06	0.14	0.13
	T2/CK2	0.93	1.00	0.92	0.08	0.11	0.10
	T3/CK3	0.92	0.92	1.00	0.07	0.12	0.08
Oxidative	T1/CK1	0.06	0.08	0.07	1.00	0.66	0.64
	T2/CK2	0.14	0.11	0.12	0.66	1.00	0.60
	T3/CK3	0.13	0.10	0.08	0.64	0.60	1.00

B

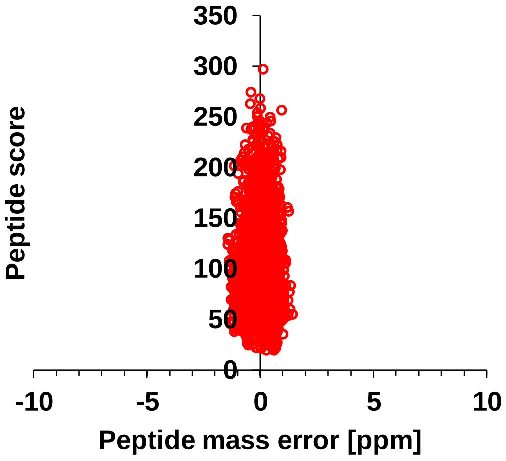

Fig. S2. Quality control of iodoTMT quantitative data.
(A) Reproducibility test between control and freezing-stressed samples used in proteomics study. The closeness of the value to 1 depicts better reproducibility of experiment. Three biological replicates were tested under both control and salt-stressed conditions.
(B) Mass error of all identified peptides. The mass error takes 0 as the central axis and concentrates within the range of less than 10 PPM (parts per million), which meets the requirements.

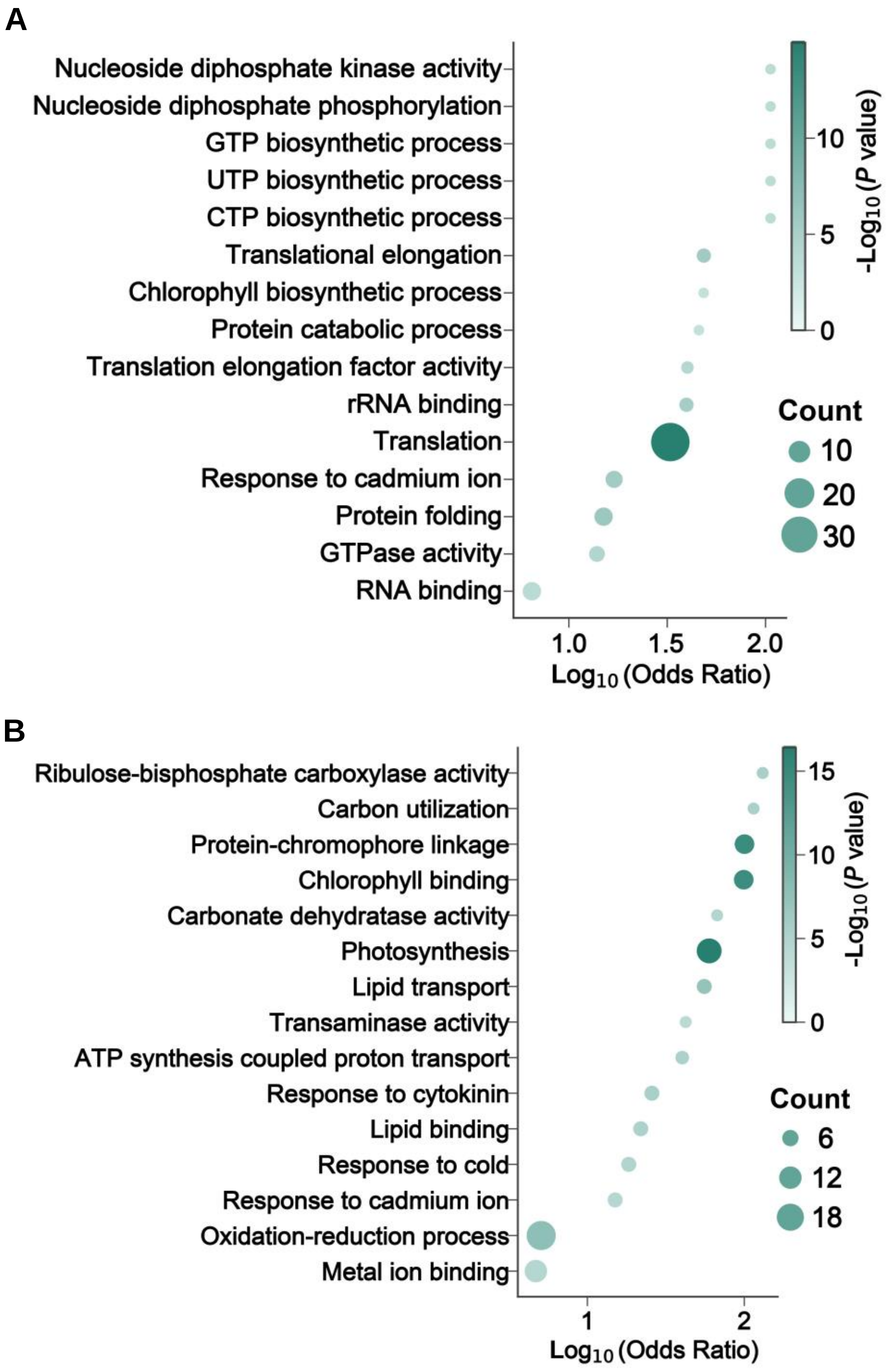

Fig. S3. GO enrichment analysis of differentially expressed proteins.

(A-B) GO enrichment analysis of DEPs, including up-regulated (A) and down-regulated proteins (B) response to freezing stress. The Log_{10} (Odds ratio) is plotted on the x-axis. The size and color of dots indicate the number of proteins and the degree of enrichment (-Log_{10}(P value), $P < 0.05$), respectively.

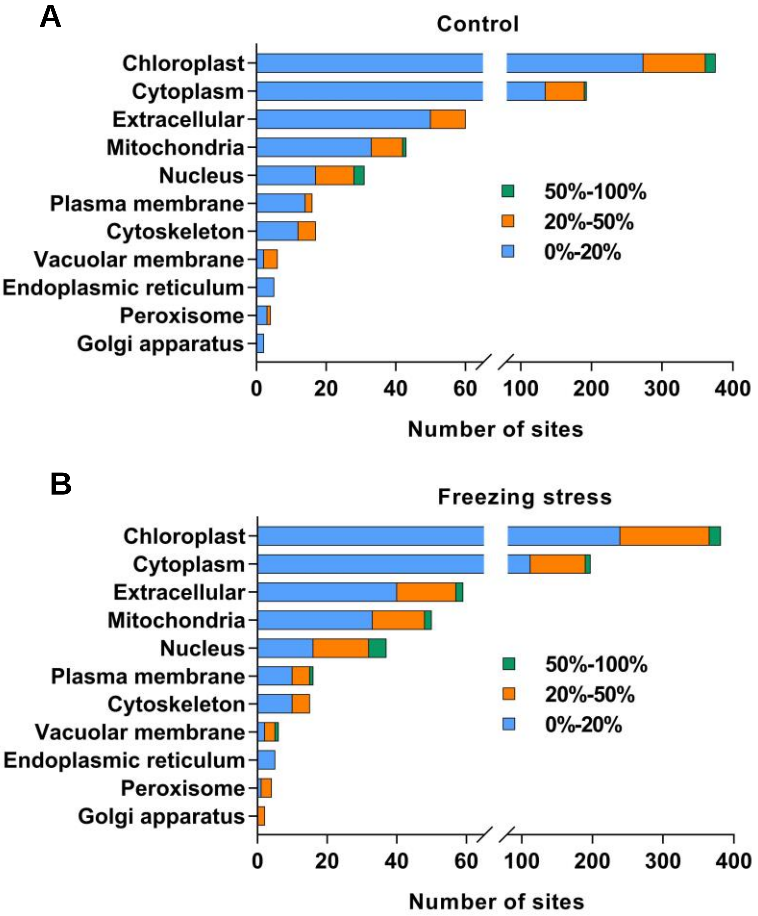

Fig. S4. %R-SOH (sulfenylated cysteines/total cysteines) distribution of sulfenylated proteins in different subcellular compartments.

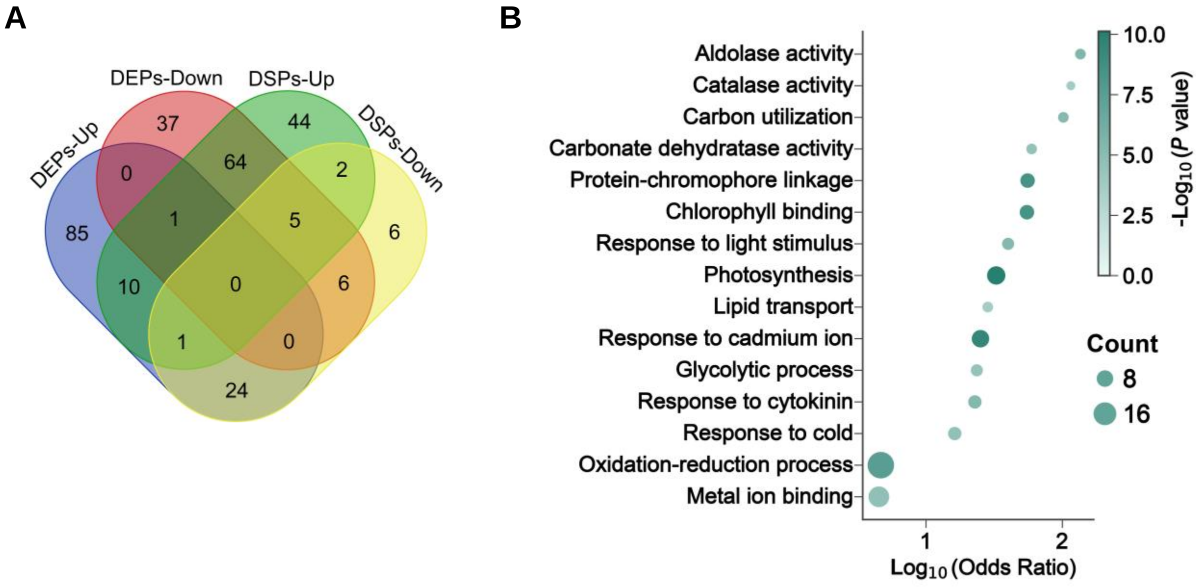

Fig. S5. Differentially expressed and differentially sulfenylated proteins.
(A) Venn diagram showing the overlapped proteins between DEPs and DSPs including up-regulated and down-regulated proteins in expression level and sulfenylation level, respectively.
(B) GO enrichment analysis of proteins with an increased sulfenylation level. The Log_{10}(Odds ratio) is plotted on the x-axis. The size and color of dots indicate the number of sulfenylated proteins and the degree of enrichment (-Log_{10}(P value), $P < 0.05$), respectively.

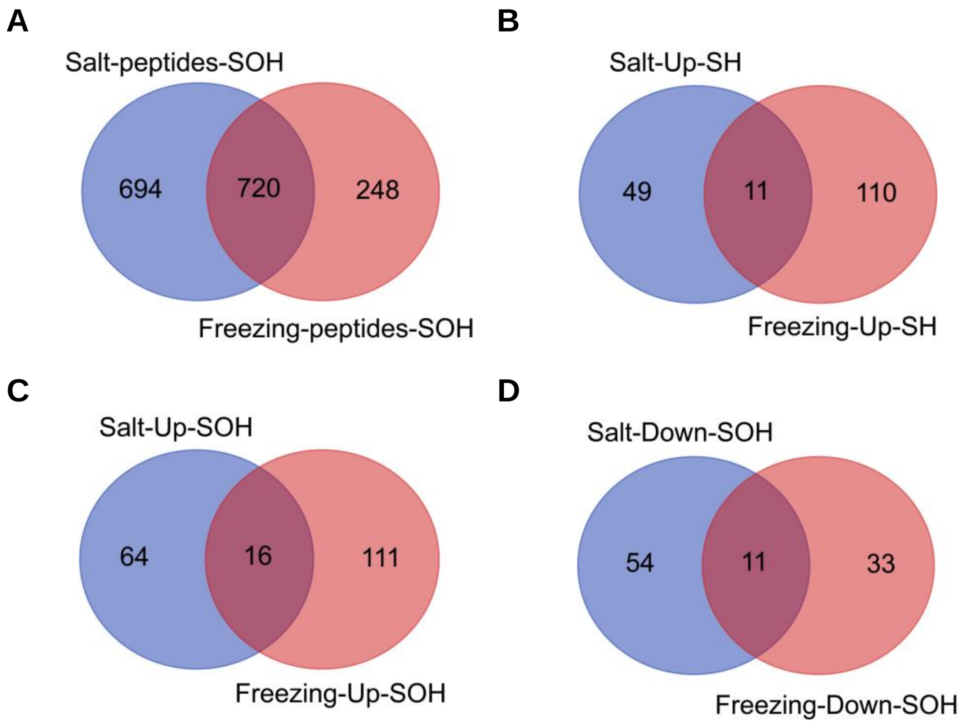

Fig. S6. Comparing of identified proteins between freezing and salt stress.
(A) The number of sulfenylated peptides identified under both salt and freezing stress.
(B) The number of proteins up-regulated in expression level under both salt and freezing stress.
(C-D) The number of DSPs identified under both salt and freezing stress, including proteins with increased (C) and decreased (D) sulfenylation level.

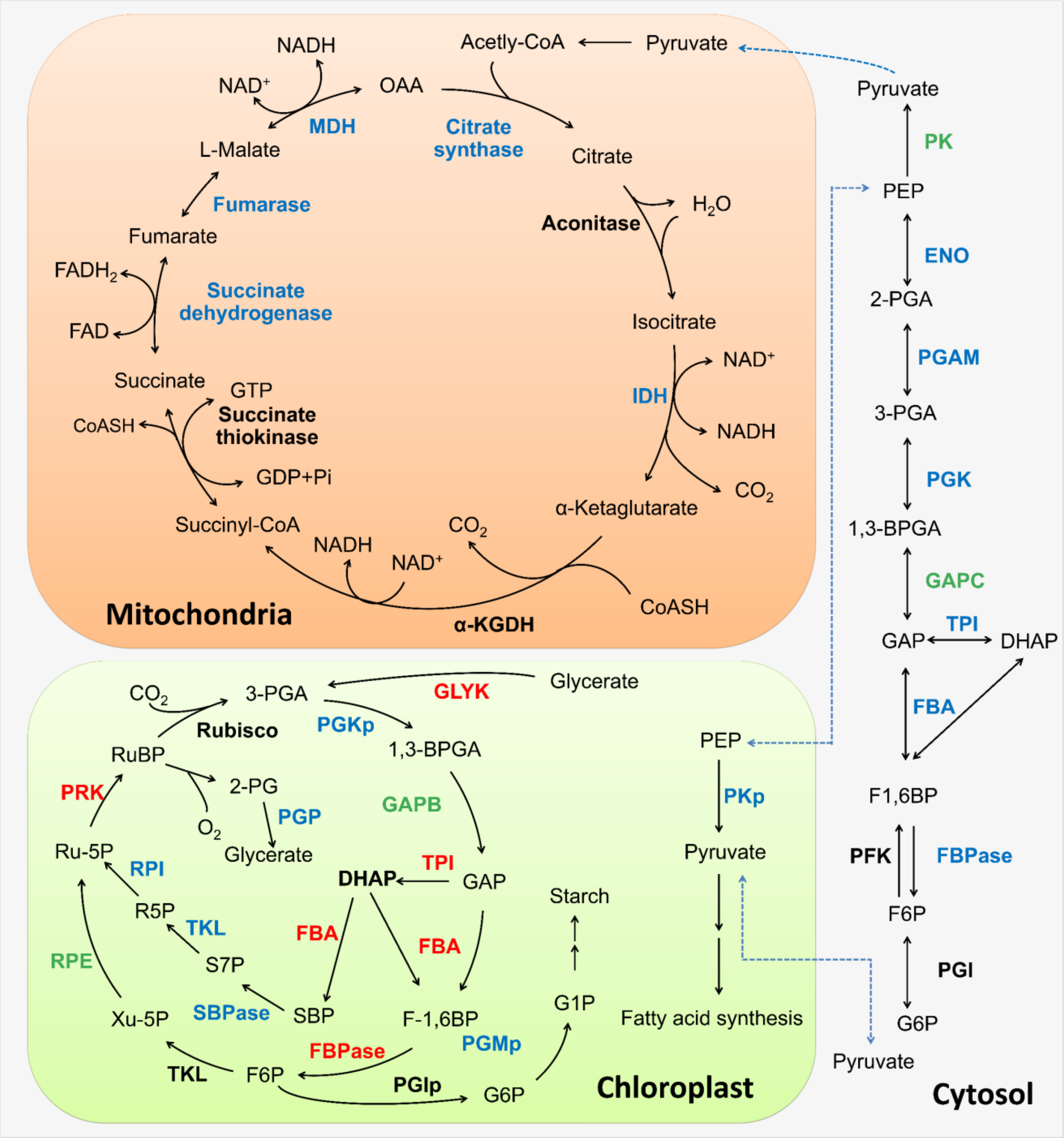

Fig. S7. Sulfenylated proteins in primary metabolic pathways. The proteins increased and decreased in sulfenylation level are in red and green, respectively. And the unaltered sulfenylated proteins are in blue. PGI, phosphoglucose isomerase; FBPase, fructose-1,6-biphosphatase; PFK, phosphofructokinase; FBA, fructose-1,6-bisphosphate aldolase; TPI, triose phosphate isomerase; GAPC, glyceraldehyde-3-phosphate dehydrogenase C; PGK, phosphoglycerate kinase; PGAM, phosphoglycerate mutase; ENO, enolase; PK, pyruvate kinase; PRK, phosphoribulo kinase; RPI, ribose 5-phosphate isomerase; TKL, transketolase; RPE, ribulose-5-phosphate-3-epimerase; GAPB, glyceraldehyde-3-phosphate dehydrogenase B; GLYK, glycerate kinases; PGP, phosphoglycolate phosphatase; MDH, malate dehydrogenase; IDH, iocitrate dehydrogenase.

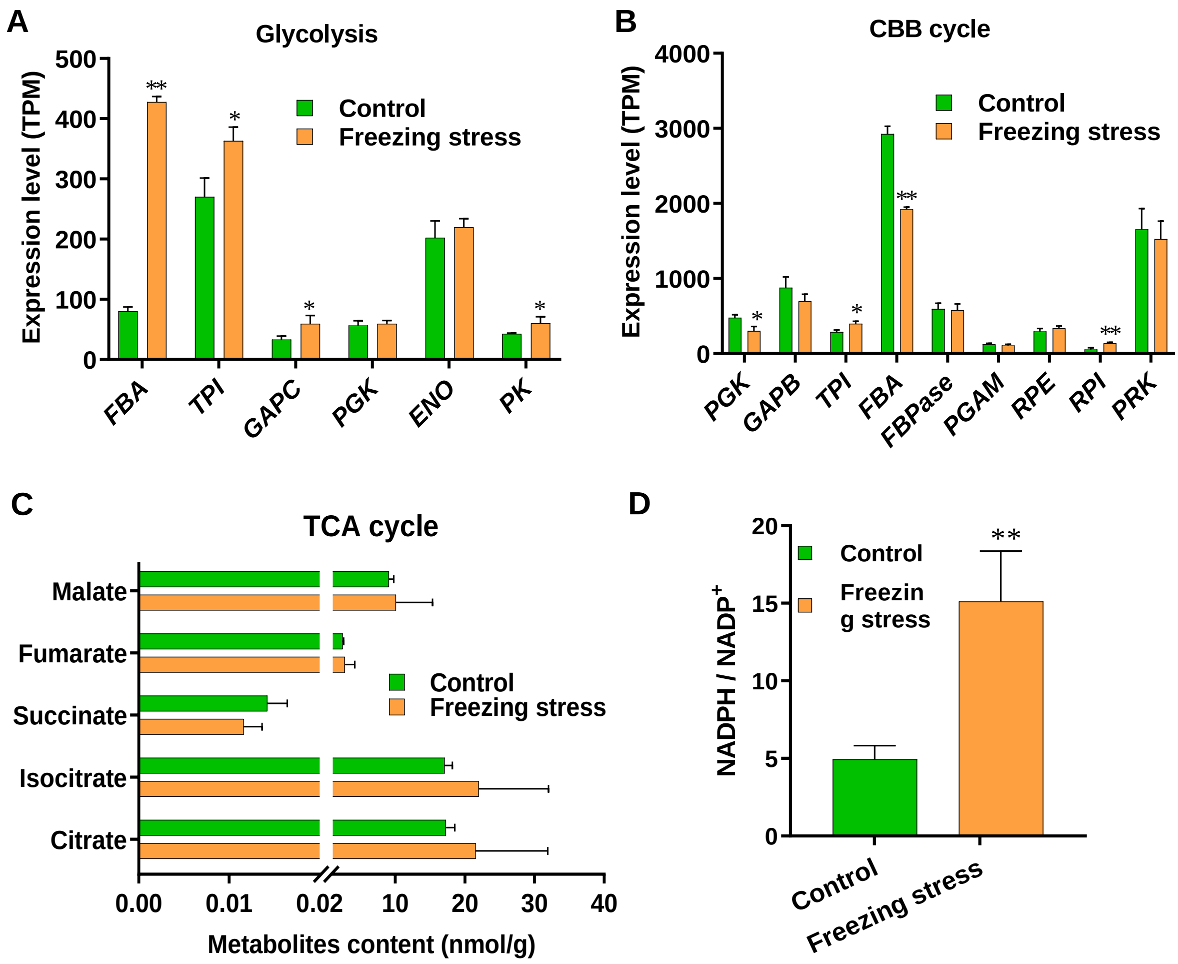

Fig. S8. Metabolite level and gene expression level of primary metabolic pathways. (A-B) Transcriptome analysis of genes involved in glycolysis pathway (A) and CBB cycle (B) by RNA sequencing. *FBA*, Fructose-1,6-bisphosphate aldolase; *TPI*, Triose phosphate isomerase; *GAPC*, glyceraldehyde-3-phosphate dehydrogenase; *PGK*, phosphoglycerate kinase; *ENO*, enolase; *PK*, pyruvate kinase; *GAPB*, glyceraldehyde-3-phosphate dehydrogenase B; *FBPase*, fructose-1,6-biphosphatase; *PGAM*, phosphoglycerate mutase; *RPE*, ribulose-5-phosphate-3-epimerase; *RPI*, ribose 5-phosphate isomerase; *PRK*, phosphoribulo kinase. Data are means ± SD (n = 3 biological replicates, *, P < 0.05, **, P < 0.01 relative to control using Student's t-test).

e e s e b i es in e in A cyc e e e ns n bi gic e ic es

e i NA NA e e ns n bi gic e ic es e i e c n using u en s es

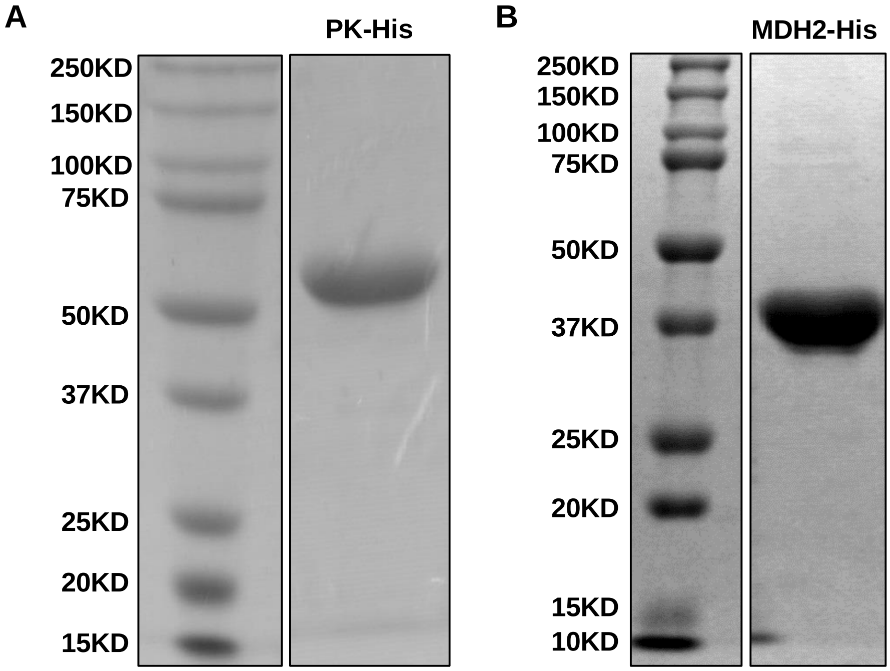

Fig. S9. Purification of recombinant protein PK (A) and MDH2 (B).
